# Spinal cord pathology is ameliorated by P2X7 antagonism in a SOD1-mutant mouse model of amyotrophic lateral sclerosis

**DOI:** 10.1242/dmm.017038

**Published:** 2014-07-18

**Authors:** Savina Apolloni, Susanna Amadio, Chiara Parisi, Alessandra Matteucci, Rosa L. Potenza, Monica Armida, Patrizia Popoli, Nadia D’Ambrosi, Cinzia Volonté

**Affiliations:** 1 Cellular Biology and Neurobiology Institute, CNR, Via del Fosso di Fiorano, 65, 00143 Rome, Italy; 2 Santa Lucia Foundation, IRCCS, Via Ardeatina, 306, 00179 Rome, Italy; 3 Department of Therapeutic Research and Medicines Evaluation, Istituto Superiore di Sanità, 00161 Rome, Italy

**Keywords:** ALS, Brilliant Blue G, Microglia, Motor neuron, P2X7

## Abstract

In recent years there has been an increasing awareness of the role of P2X7, a receptor for extracellular ATP, in modulating physiopathological mechanisms in the central nervous system. In particular, P2X7 has been shown to be implicated in neuropsychiatry, chronic pain, neurodegeneration and neuroinflammation. Remarkably, P2X7 has also been shown to be a ‘gene modifier’ in amyotrophic lateral sclerosis (ALS): the receptor is upregulated in spinal cord microglia in human and rat at advanced stages of the disease; *in vitro*, activation of P2X7 exacerbates pro-inflammatory responses in microglia that have an ALS phenotype, as well as toxicity towards neuronal cells. Despite this detrimental *in vitro* role of P2X7, in SOD1-G93A mice lacking P2X7, the clinical onset of ALS was significantly accelerated and disease progression worsened, thus indicating that the receptor might have some beneficial effects, at least at certain stages of disease. In order to clarify this dual action of P2X7 in ALS pathogenesis, in the present work we used the antagonist Brilliant Blue G (BBG), a blood-brain barrier permeable and safe drug that has already been proven to reduce neuroinflammation in traumatic brain injury, cerebral ischemia-reperfusion, neuropathic pain and experimental autoimmune encephalitis. We tested BBG in the SOD1-G93A ALS mouse model at asymptomatic, pre-symptomatic and late pre-symptomatic phases of disease. BBG at late pre-onset significantly enhanced motor neuron survival and reduced microgliosis in lumbar spinal cord, modulating inflammatory markers such as NF-κB, NADPH oxidase 2, interleukin-1β, interleukin-10 and brain-derived neurotrophic factor. This was accompanied by delayed onset and improved general conditions and motor performance, in both male and female mice, although survival appeared unaffected. Our results prove the twofold role of P2X7 in the course of ALS and establish that P2X7 modulation might represent a promising therapeutic strategy by interfering with the neuroinflammatory component of the disease.

## INTRODUCTION

Amyotrophic lateral sclerosis (ALS) is a neurodegenerative disease characterized by progressive and drastic loss of motor neurons that evolves into overall muscle impairment. Approximately 10% of individuals inherit the disease, and one of the most common forms of familial ALS is characterized by missense and gain of toxic function mutations in the gene encoding the enzyme Cu^2+^/Zn^2+^ superoxide dismutase 1 (SOD1) (Borchelt et al., 1994; Gurney, 1994). Because transgenic mice overexpressing the different mutant SOD1 proteins develop a chronic progressive motor neuron disease resembling the clinical and pathological features of ALS, they are highly exploited for investigating the mechanistic pathways of ALS and testing new potential drugs (Turner and Talbot, 2008).

Inflammation and oxidative stress play central roles in ALS pathogenesis and contribute to a vicious cycle of neurodegeneration when unhealthy motor neurons produce signals that activate microglia to release reactive oxygen species and proinflammatory cytokines (Philips and Robberecht, 2011; Sargsyan et al., 2011). Extracellular ATP binding to purinergic P2 receptors is a well-recognized neuron-to-microglia alarm molecule, and purinergic signalling is involved in several forms of neurodegeneration and neuroinflammation comprising ALS (Amadio et al., 2011; Hernández et al., 2010; Kettenmann et al., 2011; Volonté et al., 2011). Among the ionotropic P2 receptors, P2X7 in particular is implicated in diseases, such as Huntington’s, multiple sclerosis and spinal cord injury (Volonté et al., 2012), and is emerging as a ‘gene modifier’ in ALS. The receptor is upregulated in ALS spinal cord microglia in human (Yiangou et al., 2006) and rat at advanced stages of the disease (Casanovas et al., 2008). Moreover, specific activation of P2X7 dysregulates inflammatory microRNA expression (Parisi et al., 2013), exacerbates NADPH oxidase 2 (NOX2; also known as gp91^phox^) activity, reactive oxygen species production, tumor necrosis factor α (TNF-α) and levels of COX-2 and MAPKs in ALS-microglia primary cultures, with consequent toxicity towards neuronal cells (Apolloni et al., 2013a; D’Ambrosi et al., 2009), as well as providing a neurotoxic function that is mediated by astrocytes (Gandelman et al., 2010). Despite these harmful *in vitro* effects, the clinical onset of the disease is significantly accelerated in SOD1-G93A mice lacking P2X7, and the progression is worsened in both male and female mice (Apolloni et al., 2013b). This is accompanied by increased microgliosis, astrogliosis, motor neuron loss and activation, for instance, of the MAPK pathways in the lumbar spinal cord of end-stage SOD1-G93A mice lacking P2X7 (Apolloni et al., 2013b). Thus, P2X7 is likely to play a dual role in ALS.

In order to discern the multipart action mediated by P2X7, and to identify the effective time window of therapeutic intervention targeting the receptor, in the present work we have pharmacologically inhibited P2X7 in SOD1-G93A mice at different stages of the disease. We used the antagonist Brilliant Blue G (BBG), a blood-brain barrier permeable and safe drug that is already employed in clinical practice, for instance during vitreoctomy procedures (Pelayes et al., 2012). Importantly, BBG has previously provided positive results in different models of disease that are characterized by neuroinflammation, such as experimental autoimmune encephalitis (Matute et al., 2007), sciatic nerve injury (Peng et al., 2009) and Huntington’s disease (Diaz-Hernández et al., 2009).

**TRANSLATIONAL IMPACT**

**Clinical issue**

Amyotrophic lateral sclerosis (ALS) is among the most common and destructive forms of adult degeneration of motor neurons, which causes muscle impairment and eventually paralysis. Cognitive functions are generally spared in individuals with ALS, whereas muscle symptoms progressively worsen and, within 1 to 5 years from diagnosis, death occurs because of respiratory muscle failure. Approximately 10% of ALS cases are inherited and around 20% of these are linked to mutations in the gene that encodes superoxide dismutase 1 (SOD1), a key antioxidant enzyme. Inflammation and oxidative stress play key roles in ALS pathogenesis and contribute to vicious cycles of neurodegeneration, where unhealthy motor neurons produce signals able to activate microglia, which in turn release reactive oxygen species and proinflammatory factors. Extracellular ATP is a crucial microglia-to-neuron signal molecule, acting through the P2X7 purinergic receptor. Previous studies have suggested that P2X7 can function as a ‘gene modifier’ (i.e., can influence the expression of target genes) in ALS, contributing to neurodegeneration and neuroinflammation. This evidence supports the idea of testing P2X7 pharmacological antagonism as a potential therapeutic approach in ALS models.

**Results**

In this study, the authors used the SOD1-G93A mice (a well-established model of ALS that closely resembles the clinical features of the disease) and performed *in vivo* blockade of P2X7, by using the blood-brain barrier permeable and safe P2X7 antagonist Brilliant Blue G (BBG). This compound was administered to the animals at different phases of disease development in order to better clarify the role of P2X7 in the ALS-related phenotype and inflammation. The authors demonstrated that BBG administration, starting at a late pre-symptomatic phase of the disease, delays ALS onset and improves general conditions and motor performance in both male and female SOD1-G93A mice, although survival was not enhanced. Notably, the authors found that in the lumbar spinal cord of SOD1-G93A mice, treatment with BBG increased motor neuron survival and reduced microgliosis (accumulation of activated microglia), by modulating inflammatory markers such as NF-κB, NADPH oxidase 2, interleukin-1β, interleukin-10 and brain-derived neurotrophic factor.

**Implications and future directions**

This study investigates the role of P2X7 in ALS pathogenesis and provides a ‘proof of concept’ for the use of P2X7 antagonism as a strategy to ameliorate the neuroinflammatory component of ALS disease. In light of the impact that ALS has on human health and the lack of effective treatments against this disease, the pharmacological strategy proposed in this study looks promising for translation into ALS clinical trials as a potential innovative treatment to delay ALS onset and progression.

Here, we demonstrate that administration of BBG at late pre-onset significantly reduces microgliosis, modulates microglia-related inflammatory genes and enhances motor neuron survival. This is concomitant with a slightly delayed onset and an improvement of general conditions and motor performance in both male and female SOD1-G93A mice, although without effect on life span.

## RESULTS

### BBG delays the pathogenesis of ALS in SOD1-G93A mice

The object of this work is to identify the role of P2X7 inhibition *in vivo* in ALS mice, through the use of the most exploited antagonist BBG.

Our animal treatment procedure initially comprised intraperitoneal administration of BBG at 50 mg/kg of body weight to wild-type (WT) and SOD1-G93A mice for three times a week starting at late pre-onset (100 days/14 weeks of age) or onset (approximately 135 days/19 weeks). SOD1-G93A mice were considered to be at onset when they exhibited a statistically significant 10% decline of rotarod performance, with respect to WT mice. We found that although treatment with BBG (50 mg/kg) starting at onset did not modify motor impairment, at late pre-onset it improved motor performance, although only at 22 and 23 weeks of age, with respect to vehicle-treated SOD1-G93A mice (~20% increase in the BBG group with respect to vehicle). However, the median disease onset was not altered (20 weeks for both groups), and no difference between genders was found. Mice median survival was also unaffected (163 survival days for vehicle-treated mice; 161 for mice treated with BBG starting at 19 weeks; 169 for mice treated with BBG starting at 14 weeks). This was consistent with results obtained using BBG at 45 mg/kg in SOD1-G93A mice, ameliorating motor performance as assessed by rotarod and grip strength tests (Cervetto et al., 2013).

**Fig. 1.**
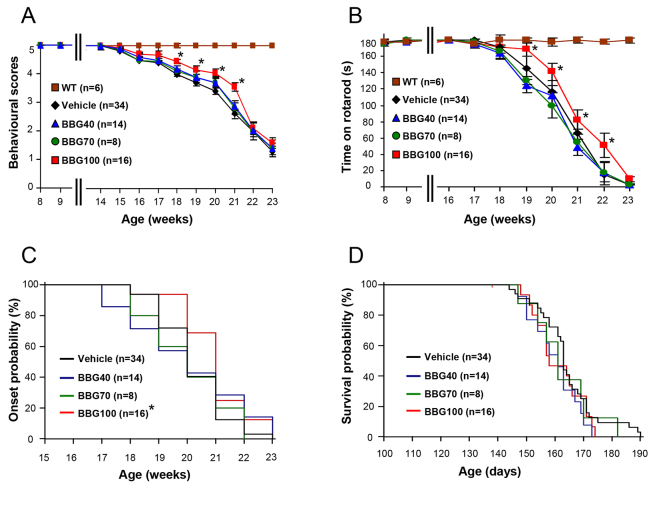
**Treatment with BBG starting at 100 days delays onset and ameliorates disease progression in SOD1-G93A mice.** (A) Behavioural scores were improved upon treatment using the BBG100 protocol (red, *n*=16), with respect to vehicle-treated mice (black, *n*=34), whereas there were no differences using the BBG70 (green, *n*=8) and BBG40 (blue, *n*=14) protocols. (B) The disease onset in SOD1-G93A mice was at 20 weeks, as determined by a decrease in rotarod performance at 15 r.p.m. The disease onset of BBG100-treated mice was delayed to 21 weeks. (C) SOD1-G93A mice showed a decline in rotarod performance with respect to WT mice (brown, *n*=6), but those treated using the BBG100 protocol showed an improvement in rotarod performance with respect to vehicle-treated mice. (D) All BBG-treated mice showed no differences in median survival with respect to vehicle-treated SOD1-G93A mice, as shown by Kaplan–Meier survival curves (158 days for the BBG100 protocol, 161 days for the BBG70 protocol and 161 days for the BBG40 protocol vs 163 days for mice treated with the vehicle). Each group of transgenic mice was statistically evaluated with respect to its proper control (vehicle group at each time point, *n*=10–12). In accordance with historical data of our colony, the behavioural, rotarod and survival curves of vehicle-treated mice were overlapping, regardless of the starting week of treatment, and thus cumulated to simplify the graphical representation. *P≤0.05.

With the aim of possibly improving BBG efficacy and, most of all, discerning its signalling mechanisms, we next tested a higher dose of the compound (250 mg/kg), also starting at earlier stages of disease, and precisely at the asymptomatic phase (40 days/6 weeks, BBG40), pre-onset phase (70 days/10 weeks, BBG70) or late pre-onset phase (100 days/14 weeks, BBG100), and BBG was administered three times a week until the end stage. Although the treatments initiated at 40 and 70 days did not significantly influence any observed parameter (Fig. 1), BBG was found to be effective when started at 100 days. In particular, as assessed by a behavioural scoring system, BBG significantly improved the overall health conditions and behavioural scores of SOD1-G93A mice between 18 and 21 weeks of age, with respect to vehicle-treated mice (Fig. 1A). As assessed by rotarod analysis, BBG improved motor performance from 20 weeks on, as compared with that of vehicle-treated mice (Fig. 1B), and also median disease onset was significantly delayed from 20 weeks in vehicle-treated SOD1-G93A mice to 21 weeks in BBG-treated SOD1-G93A mice (Fig. 1C). No differences between genders were observed at any time point, and BBG failed to prolong the median life span of SOD1-G93A mice (vehicle, 163 days; BBG40, 161 days; BBG70, 161 days; BBG100, 158 days; Fig. 1D). No significant differences in body weight were observed among all the treated groups (data not shown).

### BBG decreases microgliosis in SOD1-G93A mice at end stage

Because BBG can alleviate neuroinflammation *in vivo* (Chu et al., 2012; He et al., 2012), we next examined whether the delayed disease onset and improvement in behavioural scores and motor performance observed upon initiation of treatment with BBG at 100 days were accompanied by a modulation of the neuroinflammatory phenotype that occurred in the spinal cords of end-stage SOD1-G93A mice.

**Fig. 2.**
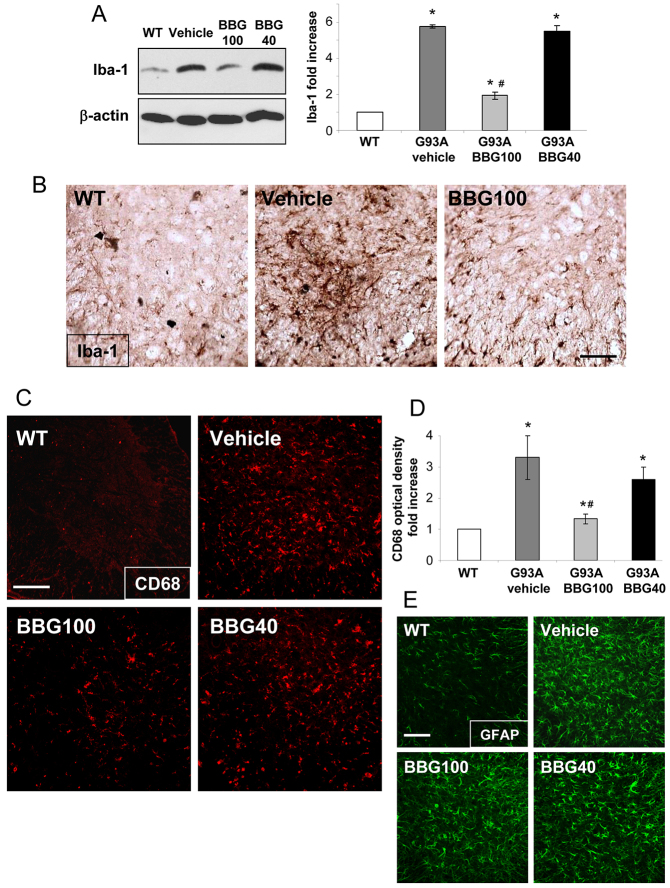
**Microgliosis but not astrocytosis is decreased in BBG-treated SOD1-G93A mice.** (A) Equal amounts of total lumbar spinal cord lysates from WT mice (6 months) and SOD1-G93A mice treated with vehicle or the BBG100 and BBG40 protocols at end stage (*n*=4) were subjected to western blotting using Iba-1 and β-actin for protein normalization. The panel on the right shows quantification of the blots relative to WT mice. (B) Spinal cord sections (L3-L5) from WT mice (6 months) and SOD1-G93A mice that had been treated with vehicle or the BBG100 protocol at end stage were stained using an antibody against Iba-1. BBG100-treated SOD1-G93A sections showed a decrease in Iba-1 labelling with respect to those from vehicle-treated SOD1-G93A mice (*n*=4–5 per group). (C) Spinal cord sections (L3-L5) from WT mice (6 months) and SOD1-G93A mice treated with vehicle or the BBG100 and BBG40 protocols at end stage were stained for CD68. (D) Quantitative analysis of CD68 in the ventral horns of spinal cord shows that CD68 optical density increases in vehicle-treated mice (dark grey) with respect to WT mice (white bar) and significantly decreases only upon treatment using the BBG100 protocol (grey bar), but not the BBG40 protocol (black bar), when compared with vehicle (*n*=4–5 per group). (E) Spinal cord sections (L3-L5) from WT mice and end-stage SOD1-G93A mice that had been treated with vehicle or the BBG100 and BBG40 protocols were stained for GFAP. Data represent means±s.e.m.; Student’s *t*-test compared with WT, *P<0.05; or with vehicle-treated SOD1-G93A, #*P*<0.05. Scale bars: 100 μm.

Before investigating the potential effects of chronic BBG administration, we proved, by using western blotting, that none of the treatments performed with SOD1-G93A mice changed the amount of the P2X7 protein in the lumbar spinal cord (Table 1). We then analysed vehicle- and BBG-treated SOD1-G93A mice for microglial markers Iba-1 and CD68 and compared them to age-matched WT mice. Consistent with behavioural features, only treatment with BBG that started at 100 days (but not at 40) decreased the expression of cytoplasmic Iba-1 protein in lumbar spinal cord by approximately 65% with respect to vehicle (Fig. 2A). The reduced expression of Iba-1 mediated by treatment with BBG initiated at 100 days was also confirmed by immunohistochemistry performed on sections of spinal cord segments L3-L5, where the abundance of immunoreactive microglia in vehicle-treated SOD1-G93A tissue was found to be drastically reduced in mice that had been treated using the BBG100 protocol (Fig. 2B). By examining L3-L5 ventral spinal cord sections through immunofluorescence and confocal analysis, we demonstrated a significant decrease (~55%) in the expression of the activated microglia antigen CD68 only in mice treated with BBG at 100 days with respect to those treated with the vehicle. Treatment with BBG at 40 days provided only a non-statistically significant reduction (*P*>0.05, Fig. 2C,D).

**Table 1.**
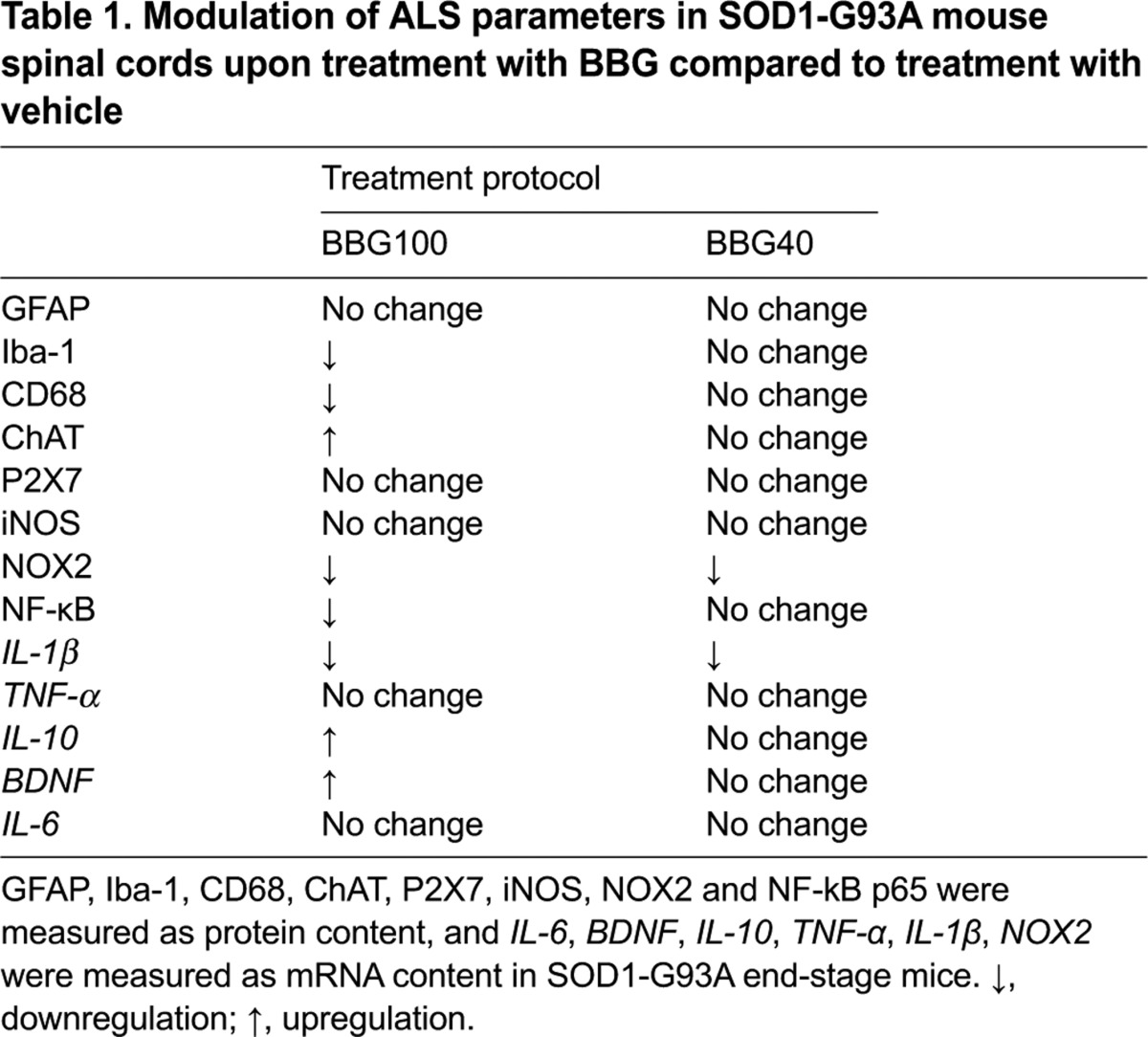
Modulation of ALS parameters in SOD1-G93A mouse spinal cords upon treatment with BBG compared to treatment with vehicle

Finally, neither BBG100 nor BBG40 significantly modified astrocytosis in L3-L5 spinal cord sections, as assessed by immunofluorescence confocal analysis (Fig. 2E) with the astrocytic marker glial fibrillary acidic protein (GFAP) – 2.7-, 2.9- and 3-fold increases were found in BBG40-, BBG100- and vehicle-treated SOD1-G93A mice, respectively, compared with WT mice. All these parameters showed no differences between genders in each group (data not shown).

### BBG affects microglia inflammatory markers in SOD1-G93A mice

We next investigated the potential modulation by BBG of several microglial inflammatory markers.

We first assessed the involvement of nuclear factor κB (NF-κB), a key mediator of neuroinflammation, which is also known to be directly stimulated in microglia by P2X7 (Ferrari et al., 1997).

**Fig. 3.**
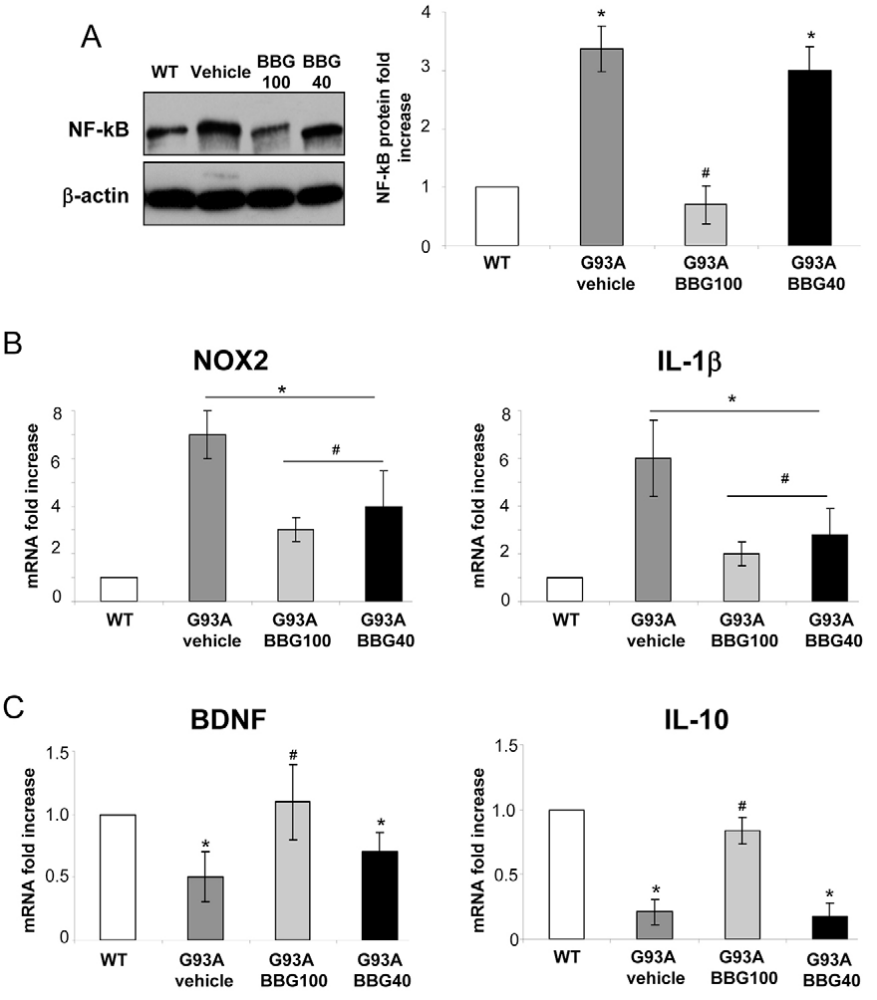
**BBG inhibits the expression of NF-κB and modulates M1 and M2 markers in SOD1-G93A mice at end stage.** (A) Equal amounts of lumbar spinal cord lysates from WT mice (6 months) and SOD1-G93A mice treated with vehicle or the BBG100 and BBG40 protocols at end stage (*n*=3) were subjected to western blotting and immunoreactions for NF-κB p65. β-actin was used for protein normalization. The chart on the right shows quantification of the blots relative to WT mice. RNA was extracted from lumbar spinal cords of WT mice (6 months) and SOD1-G93A mice treated with vehicle or the BBG100 and BBG40 protocols at end stage (*n*=4), and the expression profiles of (B) the M1 markers *NOX2* (left-hand panel) and *IL-1β* (right-hand panel) and (C) the M2 markers *BDNF* and *IL-10* were examined by using qRT-PCR. WT, white bars; vehicle, dark grey bars; BBG100, grey bars; BBG40, black bars. Data represent means±s.e.m.; Student’s *t*-test compared with WT, *P<0.05; or with vehicle-treated SOD1-G93A mice, ^#^*P*<0.05.

Consistent with modulation of microgliosis, the levels of NF-κB p65 protein increased 3.2-fold in the lumbar spinal cord of SOD1-G93A mice (vehicle) with respect to WT mice and were restored to control levels only in BBG100-treated mice (Fig. 3A). Because NF-κB upregulates the expression of several inflammatory genes (Li and Verma, 2002), we next examined typical M1 and M2 microglia inflammation-related mRNAs residing downstream of NF-κB. By performing quantitative real-time PCR (qRT-PCR) analysis on end-stage lumbar spinal cords of WT mice and SOD1-G93A mice treated with vehicle or the BBG100 and BBG40 protocols, we found that both BBG treatment protocols significantly downregulated the overexpression of typical M1 microglia markers, such as *NOX2* (stimulated 7-, 3- and 4-fold in vehicle-, BBG100- and BBG40-treated mice, respectively, compared with WT mice) and interleukin-1β (*IL-1β*; stimulated 6-, 2- and 2.7-fold in vehicle-, BBG100- and BBG40-treated mice, respectively, compared with WT mice) (Fig. 3B). Interestingly, treatment with BBG starting at 100 days, but not at 40 days, significantly enhanced, with respect to vehicle, the mRNA expression of molecules associated to the M2 phenotype, such as brain-derived neurotrophic factor (*BDNF*; 0.5-fold in vehicle-treated mice vs 1.1-fold in BBG100-treated mice with respect to WT mice) and *IL-10* (0.2-fold in vehicle-treated mice vs 0.8-fold in BBG100-treated mice, with respect to WT) (Fig. 3C). Finally, the levels of *TNF-α* and *IL-6* mRNAs, as well as inducible nitric oxide synthase (iNOS) protein, which are known to be modulated in SOD1-G93A mice, were not significantly altered upon treatment using any of the BBG protocols with respect to those of vehicle-treated mice [*TNF-α*, 0.8±0.2 and 1.1±0.2 in BBG100 and BBG40 vs vehicle (*P*>0.05); *IL-6*, 0.8±0.1 and 0.9±0.3 in BBG100 and BBG40 vs vehicle (*P*>0.05); iNOS, 0.8±0.1 and 1.1±0.2 in BBG100 and BBG40 vs vehicle (*P*>0.05); Table 1].

**Fig. 4.**
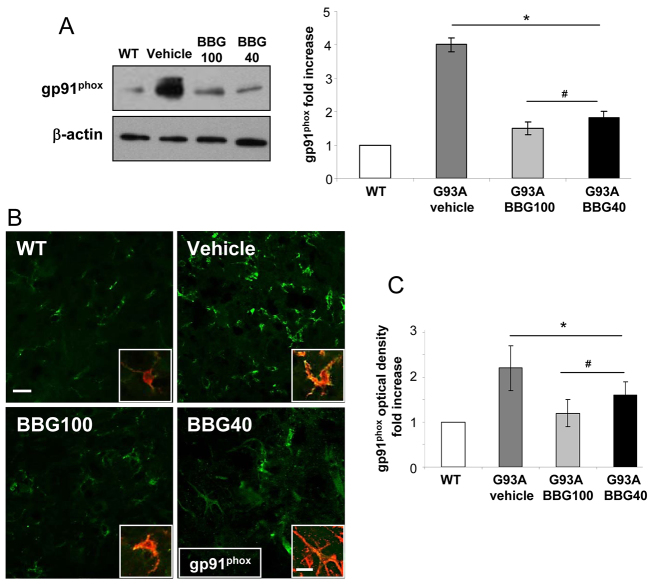
**BBG influences NOX2 expression in SOD1-G93A mice.** (A) Equal amounts of lumbar spinal cord lysates from WT mice (6 months) and SOD1-G93A end-stage mice treated with vehicle or the BBG100 and BBG40 protocols (*n*=3) were subjected to western blotting and immunoreactions for gp91^phox^ (also known as NOX2). β-actin was used for protein normalization. The chart on the right shows quantification of the blots relative to WT mice. (B) Spinal cord sections (L3-L5) from WT (6 months), vehicle, BBG100 and BBG40 SOD1-G93A mice at end stage were immunostained for gp91^phox^ (green). In the merged insets (yellow), the presence of gp91^phox^ is shown in microglial cells stained for Iba-1 (red). (C) BBG-treated mice showed a decrease in gp91^phox^ optical density, as compared with vehicle-treated SOD1-G93A mice (*n*=4–5 per group). WT, white bars; vehicle, dark grey bars; BBG100, grey bars; BBG40, black bars. Data represent means±s.e.m.; Student’s *t*-test compared with WT, *P<0.05; or with vehicle-treated SOD1-G93A mice, ^#^*P*<0.05. Scale bars: 50 μm (B); 25 μm (inset of B).

Having previously demonstrated NOX2 dysregulation by P2X7 in SOD1-G93A primary microglia (Apolloni et al., 2013a), and given the important role suggested for this superoxide-generating complex in spinal cord microglia during ALS, we further investigated the level of this protein after treatment with BBG. Western blotting analysis performed on lumbar spinal cord homogenates at the end stage demonstrated a reduction of gp91^phox^/NOX2 of about 65% in BBG100 and 60% in BBG40 mice, compared with those treated with vehicle (Fig. 4A). This was confirmed by immunofluorescence confocal analysis on L3-L5 spinal cord sections where the immunoreactive signal for gp91^phox^, which is increased in vehicle-treated SOD1-G93A mice, was instead downregulated by BBG. The previously reported colocalization of gp91^phox^ with Iba-1 (Apolloni et al., 2013b) was also confirmed (Fig. 4B).

Overall, these data indicate that only treatment with BBG starting at 100 days is able to simultaneously modulate selective markers associated with both the M1 and M2 microglia phenotypes in the lumbar spinal cord of SOD1-G93A mice.

### BBG attenuates lower motor neuron loss in SOD1-G93A mice

**Fig. 5.**
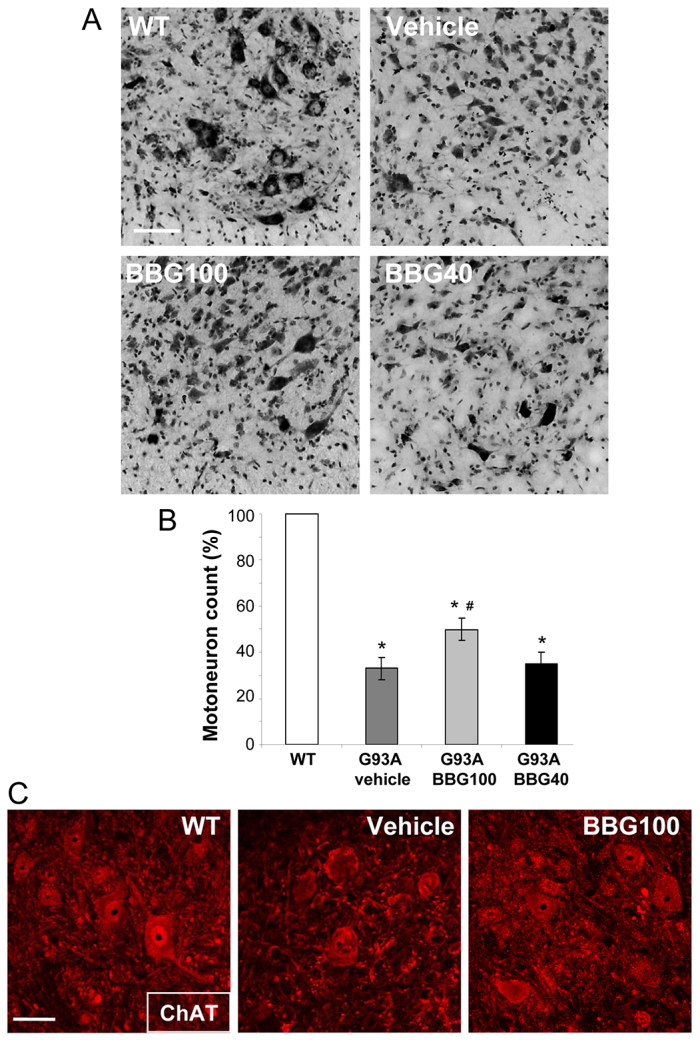
**Decreased motor neuron loss in SOD1-G93A mice treated with BBG starting at 100 days.** (A) Spinal cord sections (L3-L5) from WT mice (6 months) and SOD1-G93A mice treated with vehicle or the BBG100 and BBG40 protocols at end stage were stained with Cresyl Violet to assess motor neuron numbers. SOD1-G93A mice display an evident loss of Nissl substance with respect to WT, and this effect is attenuated in mice treated using the BBG100 protocol (B). Quantitative analysis of motor neurons in the ventral horns of spinal cord shows that vehicle- (dark grey) and BBG40-treated (black bar) SOD1-G93A mice exhibit a similar reduction of motor neuron number (35% and 37%, respectively) when compared with WT sections (white bar). BBG100-treated mice show a ~35% increase in motor neurons as compared with vehicle-treated SOD1-G93A mice (*n*=4–5 per group) (Student’s *t*-test compared with WT, ^#^*P*<0.05; or with vehicle-treated SOD1-G93A, *P<0.05). (C) Motor neurons of spinal cord sections (L3-L5) from WT mice and SOD1-G93A mice that had been treated with vehicle or the BBG100 protocol at end stage are stained with anti-ChAT. Scale bars: 100 μm (A), 50 μm (C).

In order to examine whether amelioration of motor performance and inflammatory parameters was accompanied by neuroprotection, we directly counted the number of motor neurons of WT, vehicle- and BBG-treated SOD1-G93A mice in L3-L5 spinal cord sections that had been stained with Cresyl Violet. At end stage, we observed an evident loss of Nissl substance in motor neurons of all SOD1-G93A groups. This effect was attenuated in mice that had been treated using the BBG100 protocol (Fig. 5A), where motor neuron survival was significantly improved by approximately 30% compared with vehicle-treated mice. Total motor neuron percentages were 36%, 50% and 37% compared with those of WT mice in vehicle-, BBG100- and BBG40-treated mice, respectively (Fig. 5B). No difference between genders was detected (data not shown). Motor neuron preservation in mice treated with BBG starting at 100 days, compared with that of mice treated with the vehicle, was also established through immunofluorescence and confocal analysis performed with an antibody against choline acetyltransferase (ChAT) on L3-L5 spinal cord sections from end-stage WT and vehicle- and BBG100-treated SOD1-G93A mice, as shown in Fig. 5C.

## DISCUSSION

P2X7 antagonists have shown efficacy in preventing or ameliorating various neurodegenerative features (Takenouchi et al., 2010). Among these, BBG has demonstrated effectiveness in traumatic brain injury (Kimbler et al., 2012) and cerebral ischemia-reperfusion, where it improved brain damage by directly interfering with the inflammatory response (Arbeloa et al., 2012; Chu et al., 2012). Moreover, treatment with BBG reverses chronic constriction-induced neuropathic pain through stimulating microglia activation in the sciatic nerve injury rat model (He et al., 2012). Finally, BBG improves the symptoms of Huntington’s disease and multiple sclerosis in mice (Diaz-Hernández et al., 2009; Matute et al., 2007). With the present study, we demonstrate that BBG ameliorates pathological characteristics of SOD1-G93A mice spinal cord (Table 1). In particular, when treatment was started at the late pre-symptomatic stage of the disease (100 days), BBG reduced microgliosis and proinflammatory M1 microglia markers with an increase of anti-inflammatory M2 markers, as well as motor neuron survival. This occurs along with slightly delayed onset, improvement of mouse general conditions and motor performance, and without any significant difference between genders. The lack of gender specificity is consistent with results obtained in SOD1-G93A mice that lack P2X7 (Apolloni et al., 2013b), but not with the therapeutic effect exerted by a lower dose of BBG only in male SOD1-G93A mice (Cervetto et al., 2013), belonging to a SOD1-G93A colony that, differently from ours, was showing per se gender variations.

Although we have shown here that BBG elicits beneficial effects at molecular and phenotypic levels, it does not prolong the life span of mice, further confirming that a drug with a protective effect in delaying disease onset and progression does not necessarily exert a therapeutic action in extending survival. This is consistent with the recognized multi-systemic and multi-factorial nature of ALS, and implies that P2X7 is perhaps involved only in selected pathways of the disease. However, we do not exclude that newly synthetized centrally permeable and higher affinity P2X7 antagonists with improved pharmacokinetic profiles (Bhattacharya et al., 2013) might provide more positive outcomes.

A key feature that emerged from our study is the time-dependency of BBG in showing protective effects. In contrast to the worsened SOD1-G93A phenotype obtained upon the genetic ablation of P2X7 (Apolloni et al., 2013b), a treatment started at late pre-onset (100 days), but not at pre-symptomatic phases of the disease or after onset, was indeed shown to moderate ALS progression and improve motor neuron survival. This would provide an effective therapeutic window of intervention, perhaps corresponding to the crucial switch that microglia undergo from a beneficial M2 phenotype to a detrimental M1 phenotype, and perhaps when P2X7 itself becomes crucial in modulating neuroinflammatory pathways. Although emerging studies maintain that SOD1-G93A microglia display a disease-specific transformation not classically imputable to a typical M1 or M2 phenotype (Nikodemova et al., 2013), this shift seems to occur exactly when the disease accelerates (Beers et al., 2011; Zhao et al., 2013).

In verifying the correlation between P2X7 and microglia, we further proved that the BBG100 protocol strongly reduces microgliosis in the spinal cord of SOD1-G93A mice. This is consistent with the predominant expression of P2X7 on ALS spinal cord microglia (Apolloni et al., 2013b) and with results reported in experimental animal models of neuropathic pain where P2X7 mediates microglia activation in spinal cord (He et al., 2012; Kobayashi et al., 2011). By contrast, BBG fails to affect the astrocytic, GFAP-positive, counterpart of neuroinflammation. The P2X7-microglia correlation is strengthened further by the significant downregulation of NF-κB by the BBG100 protocol, consistent with the notion that NF-κB is upregulated particularly in SOD1-G93A spinal cord microglia (Butovsky et al., 2012; Swarup et al., 2011). Activation of the NF-κB system is also known to trigger, predominantly in microglia, the expression of proinflammatory factors, such as NOX2, IL-1β and TNF-α (Pugazhenthi et al., 2013), and to regulate genes associated with the M2 phenotype, such as BDNF (Kairisalo et al., 2009) and IL-10 (Kobayashi et al., 2013). In line with this, we report a significant decrease of *IL-1β* and increase in *BDNF* and *IL-10* levels upon treatment using the BBG100 protocol in SOD1-G93A mice. NF-κB mediates NOX2 expression in spinal cord microglia (Lim et al., 2013) and, at the same time, NF-κB is known to be modulated by NOX2 in SOD1-G93A microglia (Li et al., 2011), supporting the hypothesis that oxidative stress triggers a neuroinflammatory mechanism under the regulation of NF-κB transcription. However, we have demonstrated here that NOX2 is downregulated by treatment with BBG starting at both 40 and 100 days, therefore excluding a stringent dependency between NOX2 and NF-κB under our experimental conditions. Interestingly, although starting treatment with BBG at 40 days significantly affected NOX2 expression, it did not modify disease progression. We thus suggest that blockade of NOX2 per se does not improve the ALS phenotype in SOD1-G93A mice, in accordance with a previous failure to achieve a positive effect on ALS disease progression through the use of specific NOX2 inhibitors (Trumbull et al., 2012), even though NOX2 plays a well-recognized role in ALS pathogenesis (Marden et al., 2007). As a consequence, we suggest that inhibition of selected M1 markers is not a condition that is necessary and sufficient to hamper ALS progression. Perhaps when microglia are polarized towards the M2 beneficial phenotype, as in the case of BBG100-treated mice only, the motor neuron loss is reduced and disease progression improved. Consistently, the BBG40 protocol, inhibiting M1 but not M2 markers, results in a lack of effect on motor neuron survival and disease progression.

The anti-inflammatory effects exerted by BBG *in vivo* are consistent with those reported in SOD1-G93A microglia cultures (Apolloni et al., 2013a; D’Ambrosi et al., 2009), but are in contrast with the effects of P2X7 genetic ablation in SOD1-G93A mice (Apolloni et al., 2013b). We previously reported that general conditions, symptoms onset and motor performance were worsened in SOD1-G93A mice lacking P2X7, with spinal cord gliosis and neuronal loss augmented. In addition to the dual role played by neuroinflammation and microglia in ALS (Henkel et al., 2009), our data now indicate that P2X7 might also act as a dual modifier in the disease. Consistent with low basal levels of ATP release and trophic P2X7 action that have been observed in surveilling microglia (Monif et al., 2009), or massive ATP release increasingly occurring from injured motor neurons with consequent toxic roles exerted by P2X7 in activated microglia (Kettenmann et al., 2013; Monif et al., 2010), we demonstrated that antagonism of the receptor produces positive outcomes only at a certain stage and rate of progression of the disease, thus confirming and extending to ALS the dual role of P2X7.

Although the effects of BBG have not been accompanied by an extended survival so far, P2X7 pharmacological antagonism might represent a reasonable strategy to be further exploited to understand ALS etiopathology and to ameliorate the disease progression.

## MATERIALS AND METHODS

### Mice

Adult B6.Cg-Tg(SOD1-G93A)1Gur/J mice expressing high copy number of mutant human SOD1 with a G93A substitution (SOD1-G93A) were originally obtained from Jackson Laboratories (Bar Harbor, ME, USA) and bred in our indoor animal facility. Transgenic hemizygous SOD1-G93A males were crossbred with C57BL/6 females, both maintained on the C57BL/6 genetic background.

Because a gender difference has been reported in response to pharmacological treatment with BBG in SOD1-G93A mice (Cervetto et al., 2013), we used both males and females in our experiments. Animals were housed at constant temperature (22±1°C) and relative humidity (50%) with a regular 12-hour light cycle (light 7 am–7 pm) throughout the experiments. Food and water were freely available. When animals started to fail the rotarod test (see below), macerated food was given daily for easy access to nutrition and hydration.

All animal procedures were performed according to the European Guidelines for the use of animals in research (86/609/CEE) and the requirements of Italian laws (D.L. 116/92). The ethical procedure was approved by the Animal Welfare Office, Department of Public Health and Veterinary, Nutrition and Food Safety, General Management of Animal Care and Veterinary Drugs of the Italian Ministry of Health. All efforts were made to minimize animal suffering and the number of animals necessary to produce reliable results. Transgenic progeny were genotyped by analysing tissue extracts from tail tips, as previously described (Apolloni et al., 2013b).

### BBG administration in SOD1-G93A mice

Mice were randomly assigned to groups that were treated with BBG or vehicle (NaCl 0.2% DMSO), and each group had a similar number of animals (males and females). BBG (Sigma) dissolved in vehicle was administered (250 mg/kg body weight) three times a week through an intraperitoneal injection starting at 40 (asymptomatic, BBG40), 70 (pre-onset, BBG70) or 100 (late pre-onset, BBG100) days of age, until the end stage of disease. A low dose (50 mg/kg body weight) was administrated at the beginning of symptoms onset (approximately 135 days) or 100 days of age until the end stage of the disease.

### Analysis of clinical symptoms

Transgenic animals were weighed twice a week, beginning at 40 days of age. In order to assess the general condition of mice, starting at 8 weeks of age, we used a behavioural score system with a score scale from 1 to 5 as follows: 5=healthy without any symptoms of paralysis, 4=slight signs of destabilized gait and paralysis of the hind limbs, 3=obvious paralysis and destabilized gait, 2=fully developed paralysis of the hind limbs, animals only crawl on the forelimbs, 1=fully developed paralysis of the hind limbs, animals predominantly lie on the side and/or are not able to straighten up within 30 seconds after turning them onto their back (Apolloni et al., 2013b). After reaching a score of 1, the animals were euthanized according to the guidelines for preclinical testing and colony management (Ludolph et al., 2010). Rotarod performance was tested twice a week on a rotarod apparatus (Ugo Basile 7650 model) at a constant speed of 15 r.p.m. over a maximum period of 180 seconds, starting at 8 weeks of age until animals were unable to remain on the rotarod. After a training period of 3 days, the latency to fall off was recorded as a measurement of the competence to motor function. During each test day, the animals had three trials and the best performance was recorded and included in the data analysis (Weydt et al., 2003).

### Immunofluorescence and confocal microscopy

Mice were anaesthetized by intraperitoneal injection of chloral hydrate (500 mg/kg) and transcardially perfused with 50 ml of PBS followed by 4% paraformaldehyde (pH 7.4). Tissue samples were post-fixed overnight in 4% paraformaldehyde in PBS and then cryoprotected in 30% sucrose in PBS at 4°C. Tissues were stored at −80°C. Spinal cords (L3-L5) of 30-μm thickness were cut with a frozen microtome. Double immunofluorescence analysis was performed according to the following procedure – a rectangle was drawn around the sections with a PAP pen (Sigma-Aldrich, Milan, Italy). After 1 to 2 hours of air drying, sections were blocked in PBS containing 10% normal donkey serum and 0.3% Triton X-100 for 1 hour at room temperature. Spinal cord sections were incubated with either mouse monoclonal anti-GFAP (1:10; AbD Serotech, Oxford, UK), rabbit polyclonal anti-Iba1 (1:200; Wako, Richmond, VA, USA), monoclonal rat anti-CD68 (1:100; AbD Serotech, Oxford, UK), polyclonal rabbit anti-ChAT (1:100; Millipore, MA, USA), mouse purified anti-gp91^phox^ (1:500; BD Transduction Laboratories, CA, USA), mouse anti-NOS-2 (iNOS) (1:200; Santa Cruz Biotechnology, CA, USA) in PBS, 0.3% Triton X-100 and 2% normal donkey serum for 24 hours at 4°C. Slides were washed with PBS and incubated with appropriate fluorescent-conjugated secondary antibodies for 3 hours at room temperature. The secondary antibodies were Cy3-conjugated donkey anti-rabbit immunoglobulin G (IgG) (1:100; Jackson ImmunoResearch, red immunofluorescence), Cy2-conjugated donkey anti-rabbit IgG (1:100; Jackson ImmunoResearch, green immunofluorescence), Cy2-conjugated donkey anti-mouse IgG (1:100; Alexa, Molecular Probes, Eugene, OR, USA; green immunofluorescence), or Cy3-conjugated donkey anti-rat IgG (1:100; Jackson ImmunoResearch, red immunofluorescence). PBS washes (three for 5 minutes each) were performed, and slides were cover-slipped with Fluoromount medium (Sigma-Aldrich). Immunofluorescence was analyzed by means of a confocal laser scanning microscope (Zeiss, LSM700; Germany) equipped with four laser lines: 405 nm, 488 nm, 561 nm and 639 nm. The brightness and contrast of the digital images were adjusted using the Zen software. Quantification of immunofluorescence was performed on an average of six sections of lumbar spinal cord for each animal in each group, measuring optical density by using the Zen software (Zeiss).

### Immunohistochemistry

The tissue was processed as described above and lumbar spinal cord sections from L3-L5 were mounted on poly-lysine slides. After quenching endogenous peroxidase through a 30-minute incubation with 0.3% H_2_O_2_ in PBS, sections (30-μm thick) were incubated for 24 hours in PBS 0.3% Triton X-100 and 2% normal donkey serum at 4°C with rabbit polyclonal anti-Iba1 (1:200; Wako, Richmond, VA, USA). Sections were then incubated with biotinylated donkey anti-rabbit (Jackson ImmunoResearch, West Grove, PA, USA), followed by avidin-biotin-peroxidase reactions (Vectastain, ABC kit, Vector, Burlingame, CA, USA), using 3,3′-diaminobenzidine (Sigma-Aldrich) as a chromogen. The whole ventral horn of the spinal cord was photographed at 20× magnification using an Axioskop 2 (Zeiss, Germany) microscope, and a minimum of six sections per mouse were analyzed.

### Nissl staining

The tissue was processed as described above and serial spinal cord sections (*n*=12) from L3-L5 were randomly selected and stained with 1% Cresyl Violet. Stained sections were dehydrated gradually in 50–100% alcohol, cleared in xylene and cover-slipped with Eukitt mounting medium (Sigma-Aldrich). The whole ventral horn of the spinal cord was photographed at 20× magnification using a Zeiss Axioskop 2 microscope. Large neurons, with a cell body area ≥200 μm^2^ and a definable cytoplasm with a nucleus and nucleolus (Thau et al., 2012) were then counted using Neurolucida software (MBF Bioscience, USA).

### Western blotting

Protein lysates were obtained by homogenization of mice lumbar spinal cord segments in homogenization buffer (20 mM HEPES, pH 7.4, 100 mM NaCl, 1% Triton X-100, 10 mM EDTA) supplemented with protease inhibitor cocktail (Sigma-Aldrich). After sonication, lysates were kept for 30 minutes on ice and then centrifuged for 20 minutes at 14,000 ***g*** at 4°C. Supernatants were collected and assayed for protein content with the Bradford detection kit (Bio-Rad Laboratories, Hercules, CA, USA). Separation of protein components was performed by using Mini-PROTEAN^®^ TGX™ Gels (Bio-Rad, USA) followed by transfer onto nitrocellulose membranes (Amersham Biosciences, Cologno Monzese, Italy). After saturation with ECL-Advance™ blocking agent (Amersham Biosciences), blots were probed overnight at 4°C with a specific primary antibody. Primary antibodies used were: rabbit Iba-1 (1:500; Wako); mouse gp91^phox^ (1:1000; BD Transduction Laboratories); rabbit NF-κB p65 (1:1000; Cell Signaling Technology, Beverly, MA, USA); rabbit P2X7 (1:500; Alomone Labs). After a final incubation for 1 hour with a specific horseradish peroxidase (HRP)-conjugated secondary antibody, detection was performed on X-ray film (Aurogene, Italy), using the ECL Advance western blotting detection kit (Amersham Biosciences) and signal intensity quantification by Kodak Image Station analysis software. Values were normalized with mouse anti-β-actin from Sigma-Aldrich (1:2500).

### qRT-PCR

Lumbar spinal cords were lysed in TRIzol (Life Technologies), RNA was extracted through sequential TRIzol and RNEasy Mini purification (Qiagen) and then reverse transcribed into cDNA using the Superscript Vilo cDNA synthesis kit (Life Technologies). Reactions were performed using SYBR green (Life Technologies) incorporation with gene specific primers (listed below). Relative gene expression was calculated by ΔΔCT analysis relative to *GAPDH* expression levels. Primers used – *GAPDH*: forward, 5′-CATGGCCTTCCGTGTTTCCTA-3′, reverse, 5′-CCTGCTTCACCACCTTCTTGAT-3′; *IL-1β*: forward, 5′-GCAACTGTTCCTGAACTCAACT-3′, reverse, 5′-ATCTTTTGGGGTCCGTCAACT-3′; *IL-6*: forward, 5′-GAGGATACCACTCCCAACAGACC-3′, reverse, 5′-AAGTGCATCATCGTTGTTCATACA-3′; *NOX2*: forward, 5′-TGAATGCCAGAGTCGGGATTT-3′, reverse, 5′-CCCCCTTCAGGGTTCTTGATTT-3′; *TNF-α*: forward, 5′-CTGTAGCCCACGTCGTAGC-3′, reverse, 5′-TTGAGATCCATGCCGTTG-3′; *BDNF*: forward, 5′-CGGCGCCCATGAAAGAAGTA-3′, reverse, 5′-AGACCTCTCGAACCTGCCCT-3′; *IL-10*: forward, 5′-GCATGGCCCAGAAATCAAGG-3′, reverse, 5′-GAGAAATCGATGACAGCGCC-3′.

### Statistical analysis

Data are presented as means±s.e.m. Analysis was performed with the statistical software package MedCalc (Medcalc Software, Mariakerke, Belgium). Disease onset and survival were analyzed using the Kaplan–Meier graph followed by log-rank statistics. Analysis of data was performed using ANOVA. Statistical differences between groups were verified by Student’s *t*-test. *P<0.05 was considered significant.
